# Interactive Learning Environments for the Educational Improvement of Students With Disabilities in Special Schools

**DOI:** 10.3389/fpsyg.2018.01744

**Published:** 2018-09-19

**Authors:** Rocío García-Carrión, Silvia Molina Roldán, Esther Roca Campos

**Affiliations:** ^1^Faculty of Psychology and Education, University of Deusto, Bilbao, Spain; ^2^Department of Pedagogy, Universitat Rovira i Virgili, Tarragona, Spain; ^3^Faculty of Psychology, Teacher Training and Sciences of Education, Universidad Católica de Valencia, Valencia, Spain

**Keywords:** interaction, learning, disabilities, inclusion, special schools

## Abstract

Providing an inclusive and quality education for all contributes toward the Sustainable Development Goals of the United Nations. High-quality learning environments based on what works in education benefit all students and can be particularly beneficial for children with disabilities. This article contributes to advance knowledge to enhance the quality of education of students with disabilities that are educated in special schools. This research analyses in which ways, if any, interactive learning environments can be developed in special schools and create better learning opportunities for children with disabilities. A case study was conducted with students with disabilities (*N* = 36) and teaching staff in a special school, involving interviews and focus groups. We argue that rethinking the learning context by introducing instruction models based on interaction benefit children with disabilities and provide high-quality learning and safe and supportive relationships for these students, thereby promoting their educational and social inclusion.

## Introduction

Globally, children with disabilities achieve low educational outcomes, show significantly lower rates of completion in elementary education, and face more barriers in the transition to higher levels of education, which in the long term has an impact on social exclusion and poverty in the adulthood (World Health Organization, 2011). The Convention on the Rights of Persons with Disabilities reaffirmed the international commitment to facilitate these persons the access to “an inclusive, quality, and free primary education and secondary education on an equal basis with others in the communities in which they live” (United Nations, 2007). This commitment is aligned to the global priority of ensuring inclusive and quality education for all to improve people’s lives and achieve a sustainable development.

Considering the international movement toward inclusive education, much research has focused on exploring inclusive pedagogies and teachers practice serving students with disabilities in mainstream schools (Florian and Black-Hawkins, 2011). However, students with special needs are still educated in special schools. In Europe, it occurs in varying proportions depending on the country, from a reduced percentage in Iceland, where more than 90% of these students are in mainstream schools, up to about 100% in the Walloon region of Belgium. In Spain, where this study has been conducted, the percentage of students with special needs enrolled in special schools is 17% (World Health Organization, 2011).

When compared to mainstream schools, special schools fail to provide students with special needs a maximum level of attainment in instrumental learning (language and mathematics), which seem to be explained, at least partially, by the different characteristics of both learning environments (Kocaj et al., 2014). Indeed, decades of research has provided evidence of the benefits of inclusive versus special education for students with special needs (Dunn, 1968; Calberg and Kavale, 1980; Madden and Slavin, 1983). Focusing not only on the educational placement but also on the quality of the education provided can contribute to enrich the learning opportunities of students that are not yet being educated with their non-disabled peers (Lacey and Scull, 2015). The role that psychology can have in promoting inclusive education has been claimed, being dialog a key aspect that has been emphasized (Kershner, 2016).

Particularly, in trying to identify the characteristics that can enhance the quality of special schools as a learning environment for children, it is relevant to consider research in psychology of education that has attributed a key role to interaction and dialog to explain learning processes, in an approach that has been conceptualized as the dialogic turn of educational psychology (Racionero and Padrós, 2010), and according to which interactive and dialogic learning environments maximize students’ learning opportunities and results. This approach has been developed based on the contributions of the sociocultural theory of learning initiated by Vygotsky, which explain learning and cognitive development as cultural processes that occur in the interaction with others (Vygotsky, 1962, 1978; Rogoff, 1990; Cole, 1996; Wenger, 1998). Dialog also plays a key role for learning, as it allows sharing knowledge, thoughts, and purposes (Rogoff, 1990; Bruner, 1996) and create knowledge together (Vygotsky, 1962; Edwards and Mercer, 1987; Wells, 1999; Flecha, 2000).

The social and intersubjective character of learning applies also for students with disabilities as, according to Vygotsky (1978), the students with disabilities benefit from interactive learning contexts to advance toward higher levels of learning and higher stages of development. Interactions with peers with higher levels of academic competency has been highlighted as a facilitator of greater contact of students with special needs with the general curriculum and greater learning progress of these students in regular schools (Slavin, 1996; Hanushek et al., 2003; Carter et al., 2005; Justice et al., 2014).

Recent research on learning environments that emphasize dialogical interactions and argumentation has found that these learning environments contribute to better instrumental learning outcomes of students with special needs (including vocabulary, reading, and writing) (Hand et al., 2013). Similarly, the efficacy of implementing interactive learning environments has been shown to improve prosocial behavior among elementary students (Villardón-Gallego et al., 2018). When interaction in cooperative learning is promoted, benefits are achieved both in terms of learning and social acceptance, as special education students benefit of improved self-esteem, a safer learning environment, and better learning outcomes (Jenkins et al., 2003). Students with moderate to severe intellectual disabilities gain peer acceptance, popularity, and frequency of interactions with their peers without disabilities (Piercy et al., 2002). Specific interventions based on promoting peer support have demonstrated promoting academic engagement and improvement (Carter et al., 2015, 2017), enhanced interactions and socialization (Schoger, 2006; Kohler et al., 2007; Schmidt and Stichter, 2012; Carter et al., 2015, 2017; Chung and Douglas, 2015; Lane et al., 2015; Simpson and Bui, 2017), and language development (Schmidt and Stichter, 2012).

However, scientific literature on the benefits of interactive learning environments for students with special needs are mainly focused on mainstream schools and in relation to students without special needs. Students with the most severe disabilities, who need extensive support for both access the curriculum content and non-academic skills such as interacting with others, tend to be underrepresented in the literature (Browder et al., 2014), and we still need to know which can be the effects that interactive learning environments in special segregated settings can have in special education students, to improve both their academic and social competencies.

From the perspective of providing an education of the highest quality that ensures the inclusion of all the diversity of students, and capitalizing on the benefits of interaction for learning, previous research has shown the benefits of a particular interactive learning environment, interactive groups (IG), to achieve the best levels of school success and group cohesion for all (Valls and Kyriakides, 2013; Aubert et al., 2017). Particularly, the benefits of IG for mathematics learning has been demonstrated (Díez-Palomar and Olivé, 2015; García-Carrión and Díez-Palomar, 2015). With IG, classes are organized in small heterogeneous groups of students that work together on a learning activity (mainly of instrumental content, i.e., literacy or math). Students complete the activity relying on peer interaction and mutual help, and with the support of an adult volunteer from the community that dynamize interactions. In these groups, different knowledge and abilities are shared to help everyone’s learning. IG have been identified by EU-funded research “INCLUD-ED strategies for inclusion and social cohesion in Europe from education” (FP6, European Commission, 2013) as a Successful Educational Action, because they have demonstrated to improve educational results in the different contexts where they have been implemented (Valero et al., 2018), and therefore have universal components that could be transferred to and recreated in other educational contexts (Flecha, 2015).

When implemented in mainstream schools, grouping together students with and without disabilities, IG have demonstrated to contribute to the educational inclusion of students with (and without) disabilities with positive effects both in instrumental learning and in group cohesion (García Carrión et al., 2016). However, we still do not know whether and how IG could be applied in special schools, how this implementation could respond to the challenge of achieving positive outcomes for children with special needs (Lindsay, 2016), and how this application could contribute to inclusion from special schools. In this article, we analyze the process of recreation of IG in a special school, particularly in an elementary classroom with students with disabilities learning mathematics. The aim of the study is twofold: (a) to examine how IG can be implemented in special schools and (b) to identify the improvements, if any, that this interactive learning environment has entailed for the participants. We also analyze the challenges that the school faces in this process to enhance the quality of education and opportunities of inclusion for all students.

## Materials and Methods

To carry out this research, we used the case study method, which has focused on a public special school located on the outskirts of a town in the province of Valencia, Spain. This school is distant from the urban center of the town and welcomes students from different municipalities in Valencia. The school, committed to inclusion despite being a segregated educational placement, has been working on the implementation of successful educational actions such as IG and Dialogic Literary Gatherings (García-Carrión, 2015) with its students for 2 years. The study conducted was an instrumental case study (Stake, 1995), as it allowed achieving a deep understanding of how the interactive learning environment is being implemented in the special school and how it is contributing to improve students’ educational opportunities.

### Participants

For the case study on the implementation and impact of interactive learning environments, we focused on the Primary Education group, which comprises 36 students from 6 to 14 years old with different disabilities including intellectual disability, cerebral palsy, and autism. Most of these students had participated in the interactive learning environment for three school years, and others did it for one or two school years; therefore, the number of students participating in the groups varied between school years in a range between 25 and 30 students. More detailed information about the students is presented in **Table 1**.

**Table 1:** Students’ characteristics.

		*F*	%
Gender	Male	22	61
	Female	14	39
Age	6	3	8
	7	2	6
	8	2	6
	9	4	11
	10	8	22
	11	4	11
	12	4	11
	13	5	14
	14	4	11
Disability	Intellectual disability	13	36
	Autism spectrum disorder	11	31
	Intellectual and physical	8	22
	Others	4	11
Communication	Oral	14	39
	AAC systems	5	14
	Oral and AAC systems	2	6
	Language delay	15	42
Years participating	1 year	6	17
	2 years	6	17
	3 years	23	64
	Not available	1	3
Total		36	100

Our methodological approach draws on the Communicative Methodology (Puigvert et al., 2012), an innovative approach to conduct research aimed at overcoming inequalities. Aligned with the transformative paradigm (Mertens, 2007), its main objective goes beyond to understand social and educational realities, but to discern between exclusionary and transformative elements that contribute to hinder or to overcome inequalities in the field of study. Due to the transformative orientation of this methodology, it is particularly useful in the investigation of issues that affect vulnerable groups, such as students with disabilities.

The data collection techniques used are detailed in **Table 2**, according to the timeline they were implemented.

**Table 2:** Data collection techniques.

Data collection techniques	Participants
Exploratory focus group	School teachers. The participants were 10 teachers that had been continuously implementing successful educational actions for more than one school year.
In-depth interview	School principal
Communicative focus group	Primary education teachers. The participants were three teachers of primary education students who participate in the interactive learning environment studied.
	Primary education students. The participants were four primary education students who participate in the interactive learning environment studied.

### Methodological Process

One of the researchers involved in the project was in charge of contacting and visiting the school for the data collection process. Based on a previous relationship with the teachers, who had already introduced a research-based approach in the school, the researcher contacted the school principal about participating in the study. Once obtained the school’s positive reply to participate, the researcher visited the school to give the staff additional details of the research process. Information about the school functioning, characteristics of the students, and teacher’s practice was provided to the researcher in several meetings with the staff. In a subsequent visit, the exploratory focus group with the school teachers was carried out to identify relevant topics on the development of interactive learning environments in the school. Participants agreed to provide researchers access to the relevant data for the purpose of the study. Both teachers and families were informed of the nature of the research, stressing that children’s participation was anonymous and voluntary. Likewise, it was explained that collected data would be treated with confidentiality and used solely for research purposes. Written informed consents were obtained from the principal, teachers, and the students’ parents. Ethical requirements were addressed following the Ethics Review Procedure established by the European Commission (2013) for EU research.

The topics identified in the exploratory focus group oriented the subsequent data collection to deepen in their understanding. Previous knowledge on the benefits of IG in mainstream schools identified by research was also used to guide the data collection. Finally, the interview and focus groups evolved around six topics that were subsequently used to create the categories of analysis (see **Table 3**). In the case of the focus groups with students, only the topics (a) to (d) were considered, and the focus group was conducted with the assistance of two teachers, who facilitated the communication with the students, as all of them had communication difficulties. Some of the students use regularly augmentative and alternative communication systems, while others usually communicate orally but in the focus group used pictograms to support their communication.

**Table 3:** Categories of analysis.

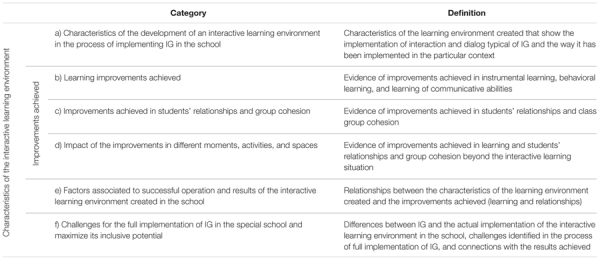

Interviews and focus groups were always conducted in the school for the participants’ convenience, with the same researcher involved in the data collection. They were audio recorded and subsequently transcribed verbatim, and notes were also taken during the students’ focus group. In all cases, the teachers and the principal had beforehand the questions for the focus groups or interviews in order to facilitate a previous reflection to the students on the object of study. Following the communicative orientation, both the interview and the focus groups were based on an intersubjective dialog between the researcher and the participants, aiming to reach an agreement on the interpretation of the reality that was object of study and therefore joint creation of knowledge (Gomez et al., 2011).

### Data Analysis

A system of categories was created deductively following the topics identified with the teachers and informed by the literature. The categorization of the data has been based on the researchers’ agreement on the category assignment of each piece of data. In **Table 3**, the categories are defined. The three coders (grounded in a content analysis approach) conducted and shared their coding and resolved any discrepancies using a consensus-based approach.

## Results

**FIGURE 1:**
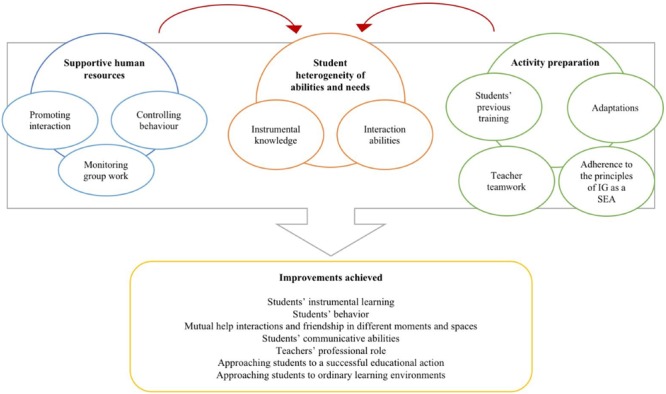
Interactive learning environment for students with disabilities: characteristics and results.

Our analysis shed light on the conditions to create an interactive learning environment in a special school with a reorganization of the existing resources and the transformations generated in the pattern of classroom interaction to improve students’ behavior and learning (see **Figure 1**). The transformative dimensions of this case study do not dismiss the complex challenges and limitations faced by professionals to create better conditions for learning and development in the context analyzed. An account on those challenges is also provided.

### Creating an Interactive Learning Environment in Special Education

In this special school, students from different elementary classrooms share together an hour session per week to work in an interactive learning environment: six small heterogeneous groups are created; each group has a different activity lasting around 15 min each focused on instrumental learning, mostly mathematics, resembling the operation of IG. In each group, four or five students work with the support of an adult, in this case a member of the school staff. Due to the specificity of the school and their students, three particular aspects are carefully designed to maximize students’ learning opportunities in this environment: (a) heterogeneity within the groups, (b) the role of the adults, and (c) the preparation of the activity.

#### Enhancing Richness of Interactions Within the Existing Diversity

Whereas all students share the characteristic of having a disability, the group of students cannot be considered homogeneous, as diversity among students is huge and difficulties and abilities vary very much. In this case, beyond the usual criteria of diversity – gender, culture, language, or level of achievement–, other criteria are taken into account to organize the groups, these are as follows: (a) students’ level of achievement and their ability to contribute to other students’ learning, (b) students’ communicative competence and ability to interact with others, and (c) students’ behavior. As teachers explain, taking into account these criteria, students are distributed in the groups based on both the difficulties they have and on what they can contribute to the others, and these contributions evolve around two key components of IG: instrumental learning and interaction. These groups are organized to operate for a whole school year. However, as their functioning is permanently assessed to ensure that learning and interaction are maximized, teachers can agree to redistribute some of the students if they observe that it might benefit the students.

So, when we started doing the groups, it was a bit different from what is normally done in the ordinary schools, we had to look for heterogeneity within the diversity that we had. Then, when we started working with these groups we decided that in each group there had to be at least one student or two that contributed knowledge to the rest, (...) another one that does not have as much cognitive level but would contribute with the procedural part, that is, we see that he or she provides many interactions, then we include one student or two with this ability, depending, and then other students who receive interactions. Then, the groups are composed like this. And it may be that also within the group, as we have many children with behavioral problems, we do not put several students with behavior problems in the same group but they are each in a group (Teacher, Focus group).

Due to the high degree of diversity, teachers’ concerns referred to how all participants would take advantage of participating in these groups. Reaching a challenging learning situation for all and avoiding that those with higher levels work below their possibilities entailed organizing the groups according to the principle of high expectations and setting the learning objectives at the highest level. Consequently, some students might be in a situation far beyond their learning possibilities at that moment; however, these cognitively challenging situations are relevant for them because those are mediated by interactions and stimulation from others that foster their learning, even if they would not achieve the highest objective set:

There is always one thing that is clear: that there are no children in the groups wasting their time, that is, something they already know to be repeating it, because if they already know it they have already learned it. Instead we look for maximum learning. So that’s why we always include students who we know they will not get the objective at that point, or maybe they will never get to, (…), which is a problem we have. But, those students are the ones that they benefit to come and to receive interactions. (…) (Teacher, Focus group)

Furthermore, the interactive environment scaffolds the students to follow their individual plans and to reach their individual goals:

Maybe he will not understand the concept “up and down,” but in the interaction within the group he places [an object] like the rest of his classmates, which is also an individual goal. So, the intention is that what is [taught] in the groups is part of his individual plan as an objective (Teacher, Focus group).

#### More Adults With More Diverse Roles to Support Students’ Learning

The adults facilitating the groups have the role to promote supportive learning interactions among student. In this school, however, the number of adults increases as compared to IG in mainstream schools as well as the diversity of their roles, to respond to students’ needs. Two adults participate in each group instead of one which is the usual in IG; their role is the same: to promote peer interactions around the learning activity. In addition, when there are students with behavior disorders, an additional adult is part of the group and takes care of behavioral problems eventually. Another professional supervises the different groups to ensure they are working effectively and provides additional support if necessary. These professionals can be the teacher, educators, or speech therapists.

#### Previous Training to Allow Students Taking Advantage of Interactions

When the session starts, students first need to know and understand the task they need to solve and then do it relying on the mutual support among students and adults. When students are not familiar with the activity, most of the time is spent in understanding what they have to do, and leave little time to work effectively. For this reason, teachers prepare the students in advance to get familiar with the task and activating the learning process, in order to make the most of the interactive situation. By observing the students, teachers realized the group experienced difficulties to perform the task when the students had not practiced the activity beforehand. It seemed crucial for the children succeed to know and to understand the task previously. So, they decided to anticipate the learning as explained:

It was an activity that was not trained. Here the school kids need to repeat the activity many times to know... Then, if they had not understood how they had to do it... The problem is that they did not know what to do, the problem was not that they did not know how to count from 1 to 10 (...) the kids that have done that before, get in front of the activity and know what they have to do, they know what it is about,. (Teacher, Focus group).

Having practiced the activity and having clearer its objective, students do not have to spend time in understanding what they have to do but directly to solve the activity interactively, and adults do not have to focus on explaining the activity but on promoting learning interactions.

What has that provoked? It has created moments in which if I know how to do it and how to solve it I am able to help you, okay? Then we have seen that there are kids that help their peers to do the activity because “I know how to do it” (Principal School, interview).

#### Carefully Designed Instruction and Systematic Evaluation

The different decisions on how to create this interactive learning environment in the school – such as the criteria of heterogeneity to organize the groups and the adults’ roles – have been made based on an agreement between the teaching staff. Teachers meet regularly to coordinate their work, including the decisions on the contents they are going to teach or practice in the groups, the activities they will propose to the students, and the most adequate materials for their students. Previously, the proposed activities are prepared by different groups of teachers that focus each on a particular block of contents (numbers, basic concepts, calculation, shape, series, etc.). The different proposals are shared and discussed in these coordination meetings, that result in a detailed planning of the sessions.

In terms of preparing the activity, adapting the material, and all the work previous to the groups, everything is established in meetings with all the staff, where the content blocks are specified: basic concepts, numbering, operations... There, the kind of activities and the specific objective are already specified: numbering from 1 to 10, numbering from 1 to 15... and it is also sequenced. In this way, the professionals develop these activities by teams, then they share them, all together, with an example of how to carry out this activity (...) For example, teachers who work on number and quantity, the whole year will be working on number and quantity, (...) and then, they present those activities in a session, and all the others presents theirs, then people say “hey, you planned to use clamps and that is very difficult for our students, you have to change it to...” (...) then you have to remember that they can use stickers, or Velcro... or think how to do it (Teacher, Focus group).

Activity adaptation is one important task of the teachers when planning the sessions. When implemented in mainstream schools, IG also contemplate the adaptation of activities and materials to allow the participation of students with special needs; the activity is the same for all the group members – otherwise interaction on the activity would be blocked – but students can accede in different ways. However, in special schools, adaptations become especially important because the diversity among students is much greater than in ordinary schools and difficulties and abilities vary very much. In the studied school, one barrier that teachers have to overcome to allow all students’ participation is the lack of literacy skills of many students, that can complicate their participation in activities mediated by written information or require writing to solve them. For this reason, activities are usually done with manipulative materials to avoid the lack of literacy skills in many students being a barrier for the learning of mathematics. The diversity of skills also includes students that are not able to speak, and others that can write but with means alternative to a pen, for instance. This diversity is considered when activities and materials are prepared. In addition, during the sessions, each participating adult has available the information on each student’s skills in order to adjust the demands and supports to them:

Within the group you can find children who write, who do not write, who use a Dymo labeler to write, or maybe they do know the numbers but as they do not write they have a Velcro adaptation... or they can speak, or they cannot speak... then depending what you find, right? Then, at each table, the children are placed and there is a sign in the table that indicates what each child can do, because not all of us know all the students. Then I come to a group and I say, look, Marcos, I know he can write, he can speak, but he cannot count or whatever. And then this gives you clues to work in the groups so that they can help each other, or to make the adaptations of material, that in many cases has to be adapted to Velcro type (…), because they know the numbers but they cannot write them, then you give them the option to solve it by taking a Velcro and placing, right? (Teacher, Focus group).

Teachers explain that these adaptations are crucial because the success of the activity depends partly on it. Therefore, they are not decided individually by a teacher, but debated and decided through agreement among the teaching team, with the ultimate objective that all students can participate and have access to the learning contents through diverse means.

Teachers also conduct systematic evaluations after each session, in which the functioning of the session and the activity are assessed. In these evaluations teachers analyze, on the one hand, that the basic principles of IG are followed (e.g., interaction, high expectations) and, on the other hand, that good results are obtained in terms of quantity and quality of interactions among students, completion of the activity and students’ learning. The teachers take notes during the sessions about the aspects that need to be improved to allow an enhancement of the interaction and learning opportunities for all students.

There is always an evaluation after the group. The person who has been supervising has a sheet where she takes notes of what she observes “I see that this group has finished the activity very soon” “I see that in this group only one knew to do the activity, therefore, we have to improve it.” Then, after the group, there is a weekly evaluation meeting and all doubts are adjusted, okay? (...) but never losing sight of the basis of the [successful] action [IG], this is always there! That is, the improvement of the interaction, the objective that children have to learn, that children have to carry out the activity. We never lose sight of that. That is the goal, then all our dialogs are aimed at the improvement of that. That is to say, all the activity has to be focused to improve learning, and everyone’s participation (School principal, interview).

### Transformation of Traditional Patterns of Classroom Interaction: Better Conditions for Caring and Supportive Learning Environments

Teachers observations and evaluation of this interactive learning environment reported a positive impact on increasing supportive and caring interactions that fostered behavioral and learning improvements among students.

#### Increasing Supportive Interactions Within the Groups and Beyond the Classroom

Creating an interactive learning environment in the classroom shifted the pattern of interaction students had engaged, so far. As teachers reported, their students had a trajectory of very individual learning, but this interactive environment facilitated the opportunities to help each other and learn together. Both the principal and the teachers agree that ‘offering help’ and ‘asking for support’ emerged as two common interaction behaviors in the group:

They help each other. I have seen this, I have seen a child being able to hold the hand of a classmate and help him point, and trying to explain it to him, with his words, very basic, but... eh... “look” “here” “there” “the number” and help him and tell him (School principal, Interview).

Teacher 1: the students themselves are already helping each other, right? and they imitate our role (…)... if they do not know for example the number 8, no? they are counting, and they get number 8, you see [one child] in the class holding another child’s hand, he is doing the sequence to count 8 and even gives to choose between two numbers, or even say “look here,” that has improved immensely.

Teacher 2: Or ask for help too, maybe someone who at one point says “do you help me?”. When you have worked a lot on the idea “ask someone to help you” or “ask someone...” well, they can also say it, that is already spontaneous in class,. (Teachers, Focus group).

Furthermore, the students in the focus group talk about mutual help, and they explain it when they are asked about what they like the most of working in these groups. In the conversation with the researcher and the teachers they increase awareness about the added value of these supportive interactions. They explain, for example, that Pilar helps Rafael taking his hand because he cannot move it alone, Fatima helps Álvaro to stick stickers, and Inés helps Wazir bringing the paper closer.

Students have learnt to help each other despite their limitations and tend to use their skills to help the others. Experiencing caring and supportive interactions in the groups helped students to move from a deficit thinking mindset toward an asset-based mindset, focusing in their strengths and opportunities rather on their problems:

Helping each other was one of the things that I saw the most difficult, because everyone has their own limitations and their own difficulties. And they have started to say “no, no, I can move my hand, I will help those who cannot move their hand “right? Or “I can speak, I will be the one who speaks about this to those who cannot speak, and I will indicate on the tablet where or how they can search.” For me that has been spectacular,. (School principal, Interview).

In this regard, according to the teachers, having the opportunity to help others has meant a change for many of them, who until that moment had only been the recipients of help. It has changed their self-concept and their beliefs about their capabilities, has empowered them, and, in some cases, it has brought changes also in their behavior, individually and as a group.

I think it has also raised their self-esteem, feeling able to help others (...) Fatima was a student with... very low expectations towards her, she had many behavioral problems, she was super absentee... (...) in the groups, she realized she is capable of helping the classmates... not always the one being helped or knowing the least or the one punished, but feeling “I am capable of doing it” has made her grow as a person, and now most of the time she is always helping her classmates, that is, she acts as a role model, helps them raise their hand, creates many interactions, asks questions, I don’t know... she has a totally different attitude than she had three years ago, (…), for me it is one of the most important cases I’ve seen how it has improved (Teacher, Focus group).

I also see the idea that “we are a group,” “we have to take this activity forward and we have to do it,” right? And I think that there has been a lot of improvement in caring relationships and classroom climate, how they talk to each other, how they respect each other, right? uh... some are also aware that “I have more capacity than him” right? And the way they treat each other, with respect, the attitude, teach us a lot. You can see it, and it is when you realize “this is the way it is, this is what solidarity means.” (School principal, Interview).

The generalization of help, care and friendship to diverse situations and moments has been identified as especially important for the children with the most severe disabilities that have very limited possibilities of interaction and in the school are recipients of basal stimulation. As a result of participating in interactive learning environments in the classroom, the number and quality of interactions that these students receive from their peers has increased:

they were all students with cerebral palsy in the same class, who did not have interactions with any other student, that is, they did not relate to each other and in the only moment they could relate was in the playground if the students approached (...). So, the tutors thought that for them it could be a moment of interactions, and since these groups have been created there are many more interactions both in the groups and in the playground moments, the students are much closer,. (Teacher, Focus group).

Some students take the lead to interact with these children and encourage other peers to follow their example, thus promoting the social inclusion of these more handicapped children within the peer group:

a child who has cerebral palsy, who at any time would have been in the playground and if the adult did not come he did not have anyone else’s interaction, (...) and after starting working in groups, for example, Ines is a girl who (…) interacts a lot with him and in the playground, makes other girls go with the basal children [highly affected children, receiving basal stimulation], that is to say, the interaction has increased a lot especially with the basal children, who were the ones who were a little more… within our special school, those who were most excluded. And then (...) a friendship group has been created and they are helping each other and calling each other (...) and you see them in the playground as they walk around with them, (…) they have changed a lot (...),. (Teacher, Focus group).

Supportive interactions have also brought the possibility for many students to “know each other” and these interactions have led to the development of new friendships. The students themselves report they listen more to each other, help each other, pay more attention to others and talk more among them. These interactions resulted in new friendships and caring relationships beyond the classroom. For instance, some students spontaneously spent their free time to play together instead of individually. The playground and the lunch time are other spaces and times in which children have been observed to help each other and build their friendship. Episodes like these show that learning interactions and mutual help have been assumed by the students as part of their everyday relationships.

Yes, I have seen it in the playground, for instance I see it a lot, how they are solidary among them, they help each other “because he is my friend” ok? In the playground (…) I think they have improved the coexistence (School principal, Interview)

#### Improvement in Students’ Behavior

Teachers have observed a clear and generalized improvement in students’ behavior in different aspects. On the one hand, the students with the most disruptive behavior, have reduced their behavioral problems, to the point that by the end of the school year there were no teacher intervention to address behavioral issues. This has been partly achieved thanks to the peer group influence, where other peers can act as role models in this interactive learning environment. The improved behavior has in turn had an impact on an increased possibility for these students to participate in the learning activity in their group and improve their instrumental learning.

I see children with many behavioral problems that paralyze them to learn anything. The child we are talking about, for example, (…), he was not able to be in a group, if you are taking off your shoes, you are getting up, you are dancing, that is... (…) I think that the progress has been huge, because seeing the others has been very important. It is now more obvious to me that what we [teachers] say to them does not have the same influence as a classmate. I can tell him “Marcos put on the shoe” 500 times and the 501st he listens to me, but maybe another classmate is the one who says that and he reacts differently (…). I don’t know, I have realized over time that they have much more power to modify that type of behavior (School principal, Interview).

Aggressive behaviors have also been reduced. There are children whose aggressive behavior has reduced in the groups sessions, while in the regular classroom activities these behaviors persist, which demonstrates the connection between the interactive learning environment and the behavioral change of these children. Again, sharing the learning activities with peers has had an impact on this change, as well as the role of adults focusing on monitoring negative behaviors.

Manuel, for example, is a student who is in the class and has behavior problems continuously, hitting... that is, very aggressive, at any time. And in the groups (…) he is in a group where there is an adult with a support role behind him, right? at first there were many behavioral problems, then the adult is a bit like modeling, right? that is to say, if perhaps he directed the hand like he was going to stretch the hair to a classmate, the adult redirected his hand from behind without interrupting in the activity and said, “do you want to ask for help?” Then, compared to how bad he behaved and the times he had to be taken out of the group (…), he has changed a lot, and in the ordinary classroom he still has behavior problems, but in the groups misconduct has diminished dramatically, (…) now he is be able to ask a classmate “do you help me?”, (…) he has done it. I think that the groups have been important and it [behavior] has been a key objective for him, to be reduced, because in class there is still misconduct and in the groups has decreased, that is, maybe you can find that he gives you a slap once, but for an entire hour that has changed a lot in Manuel (Teacher, Focus group).

The improvement of both disruptive and aggressive behavior were important learning objectives that were achieved with these children. Beyond these cases, in general, students learnt to work cooperatively, which allowed teachers to focus less on the rules to follow and more on the contents to learn.

suddenly at the end of last year there was a stability, there was calm… the behaviors were more controlled, the children were already well adapted, very used to working in this way, the professionals too, and the environmental noise dropped... and it was like: more peace, hard-working, now. (...) They arrived and suddenly they were all sitting, waiting.... With an attitude totally... and it was like... Wow! It’s fantastic! how good! while the past year was crazy... (Teacher, Focus group).

Therefore, through sustained implementation of this interactive learning environment behavioral improvements were observed and reported – leading to a learning environment free of violence, free of distractions, quiet, and focused on the activity – that are a precondition for learning.

#### Communicative Abilities and Improvement in Instrumental Learning

All students participating in the case study present communicative problems derived from their disability, although these difficulties vary between the students. Being communication a means for both learning and social relationship, having the possibility of communicating and interacting during more time and with more people, promoted an important change: from previous individualized one-to-one attention with the professionals to a multiple group interaction. Enhanced possibilities of using communication has allowed students to perform better and to be able to complete the learning tasks. Teachers recorded several improvements in instrumental learning, and these are clearly related to the contents learned interactively in the groups, which are mainly mathematical concepts.

Then we have another student who, for example, has a lot of difficulties in his speech and he had many difficulties with seriation, to do a number series, right? (...) so he is already capable of counting, sometimes helped or pointed out by his colleagues, but he is really expanding the number series. He maybe stayed in a very short number, but it has been expanding. I mean, I have seen, in general, in our students, yes, I have noticed improvements in mathematics (Teacher, Focus group).

The students themselves explained in the focus group that in these groups they “learnt more numbers” and also “learnt to help each other and ask for help,” seeing both achievements connected.

The same systematicity teachers use to evaluate the functioning of these sessions is used to evaluate students’ progress. The objectives of each students’ individual plan are permanently assessed, and they consider that these objectives are achieved when a high rate of success is obtained. Besides the achievement of learning objectives, students’ progress is also evidenced by the progressive need of less supports:

Have you noticed that the contents of your individual programming have increased in level, have improved or...?

Yes. Yes, we also have... our evaluation is: when you are working, the objective has to be achieved 8 out of 10 times, that is, there must be a lot of frequency, 8 out of 10 times that is achieved without any kind of help. And if not, then you reflect the type of help they need. And yes, we have noticed when... well, because the type of help can be total or partial, physical or verbal... or a gesture that is a signal, and then, yes, we see the progress... the type of aid they need decreases (Teacher, Focus group).

### Teachers’ Progress in Their Professional Role

Regarding teachers, the development of this interactive learning environment has been an opportunity for them to improve their students’ learning opportunities and results. It has entailed, on the one hand, an increase of teachers’ learning expectations of their students and a clear change in the way teachers speak about their students in the evaluation meetings. The language of possibility now prevails over the language of deficit, and it is especially observable for classrooms that are organized as interactive learning environments. Now teachers do not focus their discussions on their students’ limitations to learn but on what the school can do to improve their education to better adjust the educational actions to the students’ needs to enhance their progress.

regarding dialogues, for example I have attended meetings and... the way teachers talk about the students and about the way we are going to improve the action [the groups], (…) it is significant. That is, the perspective (…) Here we come to talk about the student and here we come to talk to improve the adjustment to the action [the groups] for the students to progress. I go to other types of meetings where there are no [successful educational] actions and it [the lack of progress] is the students’ fault (School, Interview)

In this regard, teachers’ training is considered to have a great importance in the school. As the objective is to recreate the best way possible IG in the school, the scientific and theoretical basis of this action is made available to teachers so that they have access to this information through original sources, and not only by informal explanations from the most experienced teachers. However, to enhance the impact on students’ learning, teachers consider the school still has room to improve finding ways to ensure that all teachers in the school have this information.

Teacher’s training (…). Because maybe you’ll find “I’ve read, I’ll tell you, I’ll explain to you...” But it is not the same what I tell you, what you interpret that (…) and what you are going to do exactly, right? I believe that all teachers’ training is necessary, that everybody knows exactly how interactive groups are made (...). What we did was to distribute articles, no? in the school there are articles related to interactive groups, their implementation... and also the training at the school level (Teacher, Focus group).

On the other hand, the development of an interactive learning environment has entailed a new and effective approach to their profession, which makes them feel they have improved as teachers. They feel that the joint work they do to plan, evaluate, and improve the intervention with their students has made them improve as professionals. According to the principal, these aspects have improved both in the students’ teachers and other teachers and educators who participate giving support.

Improvement, first the teachers’ training, argumentation of the teaching staff, the expectations of the teachers and educators because we work together, because even if they have a support role (…) [they] participate. I mean, they have improved as professionals (School principal, Interview).

Finally, the satisfaction with the work done and the impact observed in the students has made them regain hope, excitement, and meaning with their profession.

For me it has been... I don’t know, as recovering hope, and give it to our students within education. An improvement of their dignity, because we have dignified them, we have given them the word, we have given them voice and they have assumed it (School principal, Interview).

## Discussion

This case study shows that it is possible to create interactive learning contexts in special schools that lead to an improvement of the quality of education that is offered to a collective of students that too often have been educated in contexts of poor interactions and low expectations. In this school, the interactive learning environment analyzed has been created in the process of recreating IG, a successful educational action that is aligned with the main theories of learning and that has demonstrated maximize the opportunities for learning and social cohesion of diverse groups of students in the different contexts where they have been implemented. This recreation process has made available to the students most in need the educational actions that are bringing the best results in mainstream schools. As a result, students have improved their learning, their behavior, they have increased and improved their interactions with other students, have known better their peers and created friendship, and teachers have also improved as professionals.

The analysis of this interactive learning environment shows the improvements achieved are related to several conditions the teachers created for its success. The intervention is carefully designed, evaluated, and also build on teachers’ knowledge on productive dialog and interactions that foster learning and development of all students (Littleton and Mercer, 2013). Specifically, the characteristics identified that are related to the improvements achieved are as follows: an adequate training of teachers on interactive learning environments; the heterogeneous composition of the groups to promote the maximum number and diversity of interactions; the high expectations of teachers on all their students and offering them high quality education; and permanent monitoring and evaluation meetings. All these conditions reflect one main transformation in the teachers and the school: they have overcome the language of deficit to use the language of possibility. The transformation observed among the students when, for instance, they become more aware of their capabilities and use them to learn and help others learn, suggests that this language of possibility has been assumed by the students too.

However, there are several challenges teachers face in this process. The first one is the scarcity of special schools that implement IG and can be used as examples or models. Following this limitation, there is also a lack of research on the effects of IG in this type of educational contexts and on the particularities of its implementation in these schools, if any, that can enhance its success. This makes teachers feel not completely sure that they are working properly when they adapt the activity to the students’ needs while adhering as much as possible to the principles of IG and dialogic learning.

The second challenge that the school faces to recreate IG in the special school context is to guarantee the participation of all students, without any exception. Some children have serious behavioral problems, including self-inflicted injury, which makes safer for them and their peers not to participate and stay in a quieter environment. For other children, specifically some of those who have a cerebral palsy, their disability entails health problems that require permanent attention, including for instance postural changes and sleeping to prevent crisis. Teachers are concerned, on the one hand, to find ways to include those children that are not participating and, on the other hand, to make possible an active participation for those seriously affected by their disability.

Finally, the third challenge is the inclusion of volunteers from the community to facilitate the interaction in the groups. This school is physically isolated, placed 4 km far from the town. This placement, decided at a time when people with disabilities were not only segregated but also hidden, is a barrier that needs to be overcome to make the school be part of the town community life and to implement IG with family and community members as volunteers.

Despite these barriers, our findings are encouraging as they show a positive progress in the ongoing process of recreating IG in the special school context. Importantly, the evidence provided do not aim to support a defense of special schools as the preferred context to educate students with disabilities, but it opens new possibilities to improve the quality of education provided to students with disabilities in any educational context where they are placed, including those educated in special schools.

## Author Contributions

RG-C and SMR conceived the original idea and developed the theoretical framework. ERC collected the data, carried out the interviews, and observations. SMR and RG-C analyzed the data with the support of ERC. SMR wrote the first draft of the manuscript with the support of ERC. RG-C revised and edited the final version of the manuscript and supervised the project.

## Conflict of Interest Statement

The authors declare that the research was conducted in the absence of any commercial or financial relationships that could be construed as a potential conflict of interest.
